# Genetic and Molecular Mechanisms Conferring Heat Stress Tolerance in Tomato Plants

**DOI:** 10.3389/fpls.2021.786688

**Published:** 2021-12-24

**Authors:** Ken Hoshikawa, Dung Pham, Hiroshi Ezura, Roland Schafleitner, Kazuo Nakashima

**Affiliations:** ^1^Japan International Research Center for Agricultural Sciences, Tsukuba, Japan; ^2^Tsukuba Plant Innovation Research Center, University of Tsukuba, Tsukuba, Japan; ^3^Vegetable Diversity and Improvement, World Vegetable Center, Tainan, Taiwan; ^4^Faculty of Biotechnology, Vietnam National University of Agriculture, Hanoi, Vietnam

**Keywords:** climate change, abiotic stress, heat stress, molecular mechanism, vegetable, tomato

## Abstract

Climate change is a major threat to global food security. Changes in climate can directly impact food systems by reducing the production and genetic diversity of crops and their wild relatives, thereby restricting future options for breeding improved varieties and reducing the ability to adapt crops to future challenges. The global surface temperature is predicted to rise by an average of 0.3°C during the next decade, and the Paris Agreement (Paris Climate Accords) aims to limit global warming to below an average of 2°C, preferably to 1.5°C compared to pre-industrial levels. Even if the goal of the Paris Agreement can be met, the predicted rise in temperatures will increase the likelihood of extreme weather events, including heatwaves, making heat stress (HS) a major global abiotic stress factor for many crops. HS can have adverse effects on plant morphology, physiology, and biochemistry during all stages of vegetative and reproductive development. In fruiting vegetables, even moderate HS reduces fruit set and yields, and high temperatures may result in poor fruit quality. In this review, we emphasize the effects of abiotic stress, especially at high temperatures, on crop plants, such as tomatoes, touching upon key processes determining plant growth and yield. Specifically, we investigated the molecular mechanisms involved in HS tolerance and the challenges of developing heat-tolerant tomato varieties. Finally, we discuss a strategy for effectively improving the heat tolerance of vegetable crops.

## Introduction

Climate change, specifically a rise in ambient temperatures, is predicted to significantly affect plant growth and development, resulting in a devastating reduction in crop productivity, causing severe famine and limiting global food security (FAO-STAT, http://faostat.fao.org; Verisk Maplecroft, https://www.maplecroft.com; (Bita and Gerats, 2013). According to the report of the Intergovernmental Panel on Climate Change (IPCC), the accumulation of atmospheric concentrations of greenhouse gases (GHGs), such as CO_2_, N_2_O, and CH_4_, which can absorb infrared radiation reflected from the Earth's surface, was caused by the combustion of fossil energy sources and the associated GHG emissions. Changes in the atmospheric concentrations of GHGs suggested an alteration in the energy balance of our climate, causing the global surface temperature to increase by 0.3°C during the next decade and is expected to reach 1.8–4.0°C by 2100 (Jones et al., 1999; Stocker et al., 2013). During the Conference of Parties 21 (COP21) conference in Paris in 2015, governments of most countries agreed to reduce the use of fossil fuels with the ambition of a complete waiver at the end of the century, thereby attempting to limit global warming to below a 2°C increase, preferably to 1.5°C compared to pre-industrial levels. However, even if the goal of the Paris Agreement is achieved, the initiated threat of heat stress (HS) is not addressed in agriculture as HS is a major global abiotic stress factor for many crops. Due to the increased frequency of extreme weather events, including heatwaves, HS remains a threat to global agricultural production and food security. With 75% of the world's poor living in rural areas and nearly 50% of people in underdeveloped countries relying on agriculture for income, these stakeholders are likely to experience the most serious effects of climate change. In addition, a population rise to 9 billion by the year 2050 and rising food demand in rapidly growing economies, such as China and India, will require a 70% increase in food production to fulfill future needs. Increasing food production while climate change is expected to lead to tremendous crop losses is a challenge that can only be solved by more sustainable agricultural production systems using crop varieties that are more tolerant to abiotic stresses than the presently used varieties. Insights into the mechanisms allowing plants to grow and yield under stressful conditions are key to breeding more stress-tolerant varieties.

Plants, as sessile organisms, are frequently affected by adverse environmental factors, such as drought and temperatures that are hotter or colder than their optimal range. Therefore, plants adapt to stressful conditions to a certain extent. In general, when the ambient temperature is 10–15°C higher than the optimum temperature range for plant cultivation, such conditions are defined as HS (Wahid et al., 2007). HS can cause negative effects on plant morphology, development, physiology, biochemistry, and molecular pathways at all vegetative and reproductive stages. Anther and pollen development at anthesis are very sensitive to temperature fluctuations, causing failure of reproduction and fertilization processes (Warrag and Hall, 1984; Monterroso and Wien, 1990; Peet et al., 1998; Erickson and Markhart, 2002). Consequently, significant adverse effects on reproduction and fertilization processes cause a reduction in fruit set and lower quality fruit and vegetable yields (Bita and Gerats, 2013; Hasanuzzaman et al., 2013). Significant efforts by researchers and breeders are dedicated to overcoming the negative effects of HS. Plants respond to temperature fluctuations and induce short-term stress avoidance or acclimatization mechanisms, including leaf re-orientation to create space, transpiration acceleration for cooling, and alteration of membrane lipid composition (Wahid et al., 2007). At the cellular level, plants adapt to HS through various mechanisms, such as transcription, post-transcription, translation, post-translation, and regulation, at different levels, for example, in calcium, phytohormone, sugar, and lipid signaling, and in primary and secondary metabolism (Bita and Gerats, 2013). Moreover, thermotolerance is regulated by a complex transcriptome network of distinct and interconnected pathways to maintain protein homeostasis and minimize cellular damage (Keller and Simm, 2018).

Tomato (*Solanum lycopersicum*), as a fruit vegetable crop, is of immense importance to the global economy and food culture and is a popular vegetable that is produced worldwide. China is the world's largest tomato producer, followed by India and Turkey (FAOSTAT, http://www.fao.org/). Tomatoes are rich in nutrients, such as vitamin C, β-carotene, and lycopene, which have positive effects on human health (Bergougnoux, 2014). Several institutions have developed tomato genetic resources for researchers and breeders studying heat tolerance and many traits of importance. The Solanaceae Genomics Network (SGN, http://solgenomics.net/) is an online genomic database that provides essential information for researchers. In the USA, the Tomato Genetics Resource Center (TGRC) at the University of California, Davis (http://tgrc.ucdavis.edu/) is an excellent source of diverse germplasm, wild species, and core collections. In Taiwan, the World Vegetable Center (http://seed.worldveg.org) curates 8,835 tomato accessions, of which 6,676 are available on request. In Japan, genetic resources of tomato plants have been collected by the National Agriculture and Food Research Organization (NARO) Genebank (https://www.gene.affrc.go.jp) and the National BioResource Project (NBRP)-Tomato (https://tomato.nbrp.jp). In the NBRP-Tomato, over 10,000 Micro-Tom mutants, created by ethyl methanesulfonate (EMS) mutagenesis and gamma-ray irradiation, have been collected (Watanabe et al., 2007; Matsukura et al., 2008). Micro-Tom is becoming a model plant for studying both fruit production and tolerance to various abiotic and biotic stresses (Ezura, 2016). Researchers can access information regarding this mutagenic line through the online database TOMATOMA (http://tomatoma.nbrp.jp/index.jsp) (Saito et al., 2011; Shikata et al., 2016).

Tomato plants are often exposed to temperature fluctuations during cultivation, and HS significantly affects reproduction and fertilization, leading to crop failure and a decrease in the quantity and quality of harvested fruit (Prasad et al., 1999; Sato et al., 2000). The morphological and physiological changes in response to HS in tomatoes are different among entries or accessions, at different development stages, and with varying HS exposure periods. These changes are not only detected in vegetative organs, such as leaves (Zhou et al., 2017), but also in reproductive organs, such as flowers and gametophytes (Firon et al., 2006). Firon et al. (2006) reported that the relationship between pollen viability and fruit set in tomatoes was detected under HS conditions. Pan et al. (2019) reported that the alteration of flower structure, such as stigma exertion, was associated with jasmonate (JA) signaling and other plant hormone pathways, resulting in low fruit setting.

We reviewed the effects of high temperatures on tomato plants to address key processes determining plant growth and yield. This review focuses on the molecular mechanisms, the morphological and physiological mechanisms contributing to HS tolerance, and the challenges in developing heat-tolerant vegetable varieties.

## Morphological and Physiological Processes in Tomato Plants Under HS

**Figure 1:**
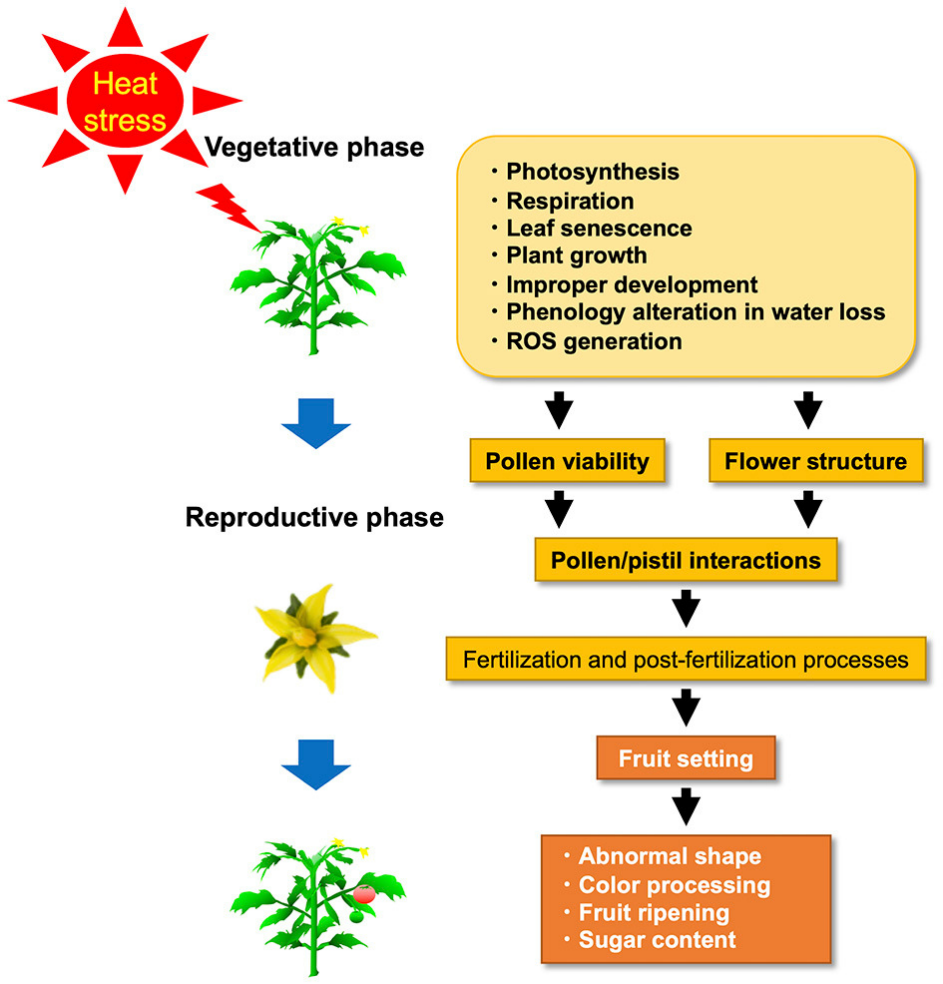
Adverse effects of heat stress (HS) on the vegetative and reproductive phase in tomatoes.

Plant response to HS varies according to developmental stage, species, genotype, and the timing of **HS** events (Firon et al., 2006; Barnabás et al., 2008; Sakata and Higashitani, 2008; Shanmugam et al., 2013; Sharma et al., 2014) (Figure 1). HS resistance is genetically diverse (Ayenan et al., 2019; Bineau et al., 2021). Since their physiological mechanisms are equally diverse, we will first explain the physiological mechanisms and then explain the genetic diversity of HS resistance for breeding. Under HS, plants exhibit many physiological responses, such as abscission and senescence of leaves, growth inhibition of the shoots and roots, and fruit damage, resulting in a substantial decrease in plant productivity (Figure 1) (Vollenweider and Günthardt-Goerg, 2005). Extreme HS affects performance and crop quality characteristics. The productivity decrease under HS has been attributed to decreased assimilatory capacity associated with reduced photosynthesis caused by altered membrane stability, enhanced maintenance respiration costs, and a reduction in radiation use efficiency (Zhang et al., 2006; Reynolds et al., 2007; Hasanuzzaman et al., 2013). At the beginning of cultivation, reduced germination percentage, reduced plant emergence, abnormal seedlings, poor seedling vigor, and reduced radicle and plumule growth of germinated seedlings are major impacts of HS and have been documented in various cultivated plant species (Toh et al., 2008; Kumar et al., 2011; Piramila et al., 2012). When tomato plants are cultivated at 42°C, they sustain severe damage at various stages of development, including seed germination, vegetative and reproductive growth, and fruit setting (Wahid et al., 2007).

In general, the intensity, duration, and rate of temperature alteration during the growth and development of tomatoes are the main factors for evaluating the influence of HS (Wahid et al., 2007). During primary synthesis and respiration processes, leaves retain stomata machinery, which regulates gas exchange and water vapor between the atmosphere and the intracellular space, resulting in an adaptation to changes in the cultivation environment (Negi et al., 2008). An increase in CO_2_ concentration inhibits the opening and closing of stomata on stomatal apertures (Medlyn et al., 2001; Hashimoto et al., 2006; Ainsworth and Rogers, 2007; Ji et al., 2015). The temperature on the leaf surface influences stomatal density and status; for example, the opening and closing of stomata form a complex network that controls gas exchange and water vapor to adapt to abiotic stresses (Valladares and Pearcy, 1997; Reynolds-Henne et al., 2010). Tomato plants of the cultivar Campbell 28, when heat treated (45°C), had increased stomatal conductance compared to plants under control conditions (25°C), indicating that stomatal closure did not control the reduction in CO_2_ (Camejo et al. 2005). Increases in stomatal conductance have been reported in plants exposed to HS (Radin et al., 1994; Zhou et al., 2015), while others have found that stomatal conductance is significantly reduced (Weston and Bauerle, 2007; Neill et al., 2008; Lahr et al., 2015; von Caemmerer and Evans, 2015). It is known that salicylic acid (SA) auxin, cytokinin, ethylene (ET), brassinosteroids (BRs), and JA regulate stomatal function, while abscisic acid (ABA) is not involved (Miura and Tada, 2014).

Heat stress adversely affects respiration and photosynthesis, leading to a shortening of the life cycle and a significant decrease in plant productivity (Barnabás et al., 2008). At the beginning of HS, the response is expressed through structural alterations in chloroplast protein complexes and reduced enzyme activity (Bita and Gerats, 2013), followed by damage to the cell membrane and the organization of microtubules. The cytoskeleton can also be damaged, because HS negatively influences membrane permeability, causing alterations in cell differentiation, elongation, and expansion (Smertenko et al., 1997; Potters et al., 2009). The retention of cellular membrane function is essential for sustainable and stable photosynthetic and respiratory continuity under HS (Chen et al., 2012). Some researchers have reported that swelling and aberration of grana stacks occur on photosynthetic membranes, resulting in associated changes in energy allocation to photosystems and ion leakage from leaf cells (Wahid and Shabbir, 2005; Allakhverdiev et al., 2008).

The negative effect of HS on chlorophyll and the photosynthetic apparatus results in the overproduction of reactive oxygen species (ROS), which are involved in responses to biotic and abiotic stresses (Vara Prasad et al., 2000; Shi et al., 2015). HS reduces photosynthesis and respiratory activity by increasing chlorophyllase activity and reducing the number of photosynthetic pigments (Todorov et al., 2003; Sharkey and Zhang, 2010). An increase in the concentration of ROS was not only associated with programmed cell death (PCD) but also with various metabolic reactions, such as DNA damage, enzyme activity impairment, lipid peroxidation in cellular membranes, carbohydrate oxidation, protein denaturation, and the breakdown of pigments (Bose et al., 2014). Hydrogen peroxide (H_2_O_2_) is one of the main ROS components produced by plants and fruit tissues under control and HS conditions. In plants, H_2_O_2_ functions not only as an essential signal that upregulates antioxidant enzyme activities but also mediates ABA-induced stomatal closure to promote stress tolerance (Hu et al., 2005). In addition, hydrogen peroxide accumulates and enhances the thermotolerance of plants when they are treated with low concentrations of SA (Horváth et al., 2007).

Ionic leakage is related to ROS accumulation under stress conditions (Demidchik et al., 2014). Drought stress has been found to result in increased ion leakage in drought-sensitive tomato entry (Thirumalaikumar et al., 2018). There are two popular methods for measuring ion leakage to estimate the heat tolerance in plants: (i) the common ion leakage measurement based on the total electrical conductivity released before and after heating and (ii) the estimation of basal heat tolerance based on the cell suspension or the gradual (linear) heating of plant segments (Ilík et al., 2018). Total ionic leakage is among the most important factors in determining plant responses to abiotic and biotic stresses, as it is associated with stress-induced injury related to PCD in plants (Zhu, 2016).

Antioxidant defense plays an important role in the response of tomato plants to various abiotic stresses. **HS** causes serious damage to antioxidant enzymes function; therefore, tomato plants are required to regulate SA and activate other biochemical pathways to enhance heat tolerance (Jahan et al., 2019). ROS acts as a transduction signal of heat tolerance; hence, superoxide dismutase (SOD) and ascorbic acid peroxidase (APX) are involved in the antioxidant defense mechanism in tomato plants in response to the negative effects of high temperature (Zhou et al., 2019) (Table 1).

**Table 1:** Key genes related to heat stress (HS) mechanisms are introduced in this review.

**Gene/locus symbol**	**Origin**	**Defined function**	**Related trait/phenotype**	**References**
*SOD*	Tomato	Antioxidant enzyme	Antioxidant defense	Zhou et al., 2019
*APX*	Tomato	Antioxidant enzyme	Antioxidant defense	Zhou et al., 2019
*SENU3*	Tomato, Pepper	Senescence-associated cysteine proteinase Vacuolar localization protein	Leaf senescence	Drake et al., 1996; Xiao et al., 2014
*RbcL*	Potato	Carbon assimilation and fixation	Leaf senescence	Enyedi and Pell, 1992; Wang et al., 2015
*HsfA1 a, b, c, d*	*Arabidopsis*	Transcriptional activators to HS	Transcription regulatory network	Liu et al., 2011; Ohama et al., 2017
*HsfA1*	*Arabidopsis*	Master regulator of HSR	Transcription regulatory network	Yoshida et al., 2011
*HsfB1, HsfB2b*	*Arabidopsis*	Transcriptional repressors	Transcription regulatory network	Ikeda et al., 2011
*HsfA1d*	*Thellungiella salsuginea*	HS-responsive gene expression, Temperature-dependent repression (TDR) domain	Transcription regulatory network	Higashi et al., 2013; Ohama et al., 2017
*DREB2A*	*Arabidopsis*	Transcriptional activators to HS	Transcription regulatory network	Ohama et al., 2017
*HsfA1, a, b, c, e*	Tomato	Transcriptional activators to HS	Transcription regulatory network	El-Shershaby et al., 2019
*HsfA1b*	Tomato	Later response gene in transcription regulatory network	Transcription regulatory network	El-Shershaby et al., 2019
*ERF.C1/F4/F5*	Tomato	Ethylene-responsive transcription factors	HS regulation	Balyan et al., 2020
*HSFA7, HSFA6b, HSFA4c, HSFB1, HSFB2b*	Tomato	Downstream targets of HSFA	Transcription regulatory network	Rao et al., 2021
*HSPs*	*Arabidopsis*	Chaperone proteins regulating the folding and accumulation of proteins, localization, and degradation	Transcription regulatory network	Kotak et al., 2007; Qu et al., 2013
*AP1*	*Arabidopsis*	Class A activity	Flower morphology	Wellmer et al., 2014
*AP3, PI*	*Arabidopsis*	Class B activity	Flower morphology	Wellmer et al., 2014
*AG*	*Arabidopsis*	Class C activity	Flower morphology	Wellmer et al., 2014
*STK, SHP1, SHP2*	*Arabidopsis*	Class D activity	Flower morphology	Wellmer et al., 2014
*SEP1, SEP2, SEP3, SEP4*	*Arabidopsis*	Class E activity	Flower morphology	Wellmer et al., 2014
*AGL6*	*Arabidopsis*	MADS-box transcription factor	Flower morphology	Wellmer et al., 2014
*TTS, TGL11*	Tomato	Pistil-specific expression	Flower morphology	Müller et al., 2016
*TAP3, TM6, PI*	Tomato	Class B activity	Flower morphology	Müller et al., 2016
*AGL6*	Tomato	MADS-box transcription factor, fruit parthenocarpy	Flower morphology, Fruit parthenocarpy	Klap et al., 2017
*CLV*	Tomato	Signal peptide, shoot, and floral meristem regulation	Shoot and floral meristem	Somssich et al., 2016; Fletcher, 2018; Quinet et al., 2019
*WUS*	Tomato	Homeodomain transcription factor, shoot and floral meristem regulation	Shoot and floral meristem	Somssich et al., 2016; Fletcher, 2018; Quinet et al., 2019
*ELF3*	*Arabidopsis*	Transcriptional repressor	Auxin-dependent primordia production	Jones et al., 2021
*TAG1, TAGL1*	Tomato	MADS-box transcription factor	Fruit size	Gimenez et al., 2016
*ZjDA3*	*Ziziphus jujuba*	ubiquitin-specific protease	Fruit size	Guo et al., 2021
*CCS52A, WEE1*	Tomato	Cell cycle switch protein	Fruit size	Gonzalez et al., 2007; Mathieu-Rivet et al., 2010
*miRNA172*	Tomato Apple	miRNA	Fruit size	José Ripoll et al., 2015; Yao et al., 2016
*FAS, LC*	Tomato	Flattening and fruit locule number	Fruit size	Rodríguez et al., 2011
*SUN, OVATE*	Tomato	Fruit elongation	Fruit size	Rodríguez et al., 2011
*LIN5*	Tomato	Cell wall invertase	Fruit sugar	Fridman et al., 2000, 2004
*SUT or SUC*	*Arabidopsis*, Tomato, Potato	Sucrose transporter	Fruit sugar	Barker et al., 2000; Weise et al., 2000; Hackel et al., 2006
*SlVPEs*	Tomato	Vascular processing enzymes, negative regulators	Fruit sugar	Ariizumi et al., 2011

It has been reported that leaf senescence is accelerated by HS during cultivation. Leaf senescence genes are correlated with PCD and are regulated by multiple levels of chromatin structure, transcription, post-transcription, translation, and post-translation (Woo et al., 2013) (Table 1). Leaf senescence genes are also interconnected with other genes responsible for responding to abiotic and biotic stresses. Senescence upregulated 3 (*SENU3)* is a ubiquitous cysteine protease (CP) that is associated with vacuolar senescence in pepper (Drake et al., 1996; Xiao et al., 2014). Another gene involved in leaf senescence is the rubisco large subunit (*RbcL*), which is in the chloroplast DNA and functions as a key enzyme for carbon assimilation and fixation (Enyedi and Pell, 1992; Wang et al., 2015). *RbcL* expression is regulated in response to environmental changes (Xu and Tabita, 1996).

## Molecular Mechanism for Thermotolerance in Tomatoes

**Figure 2:**
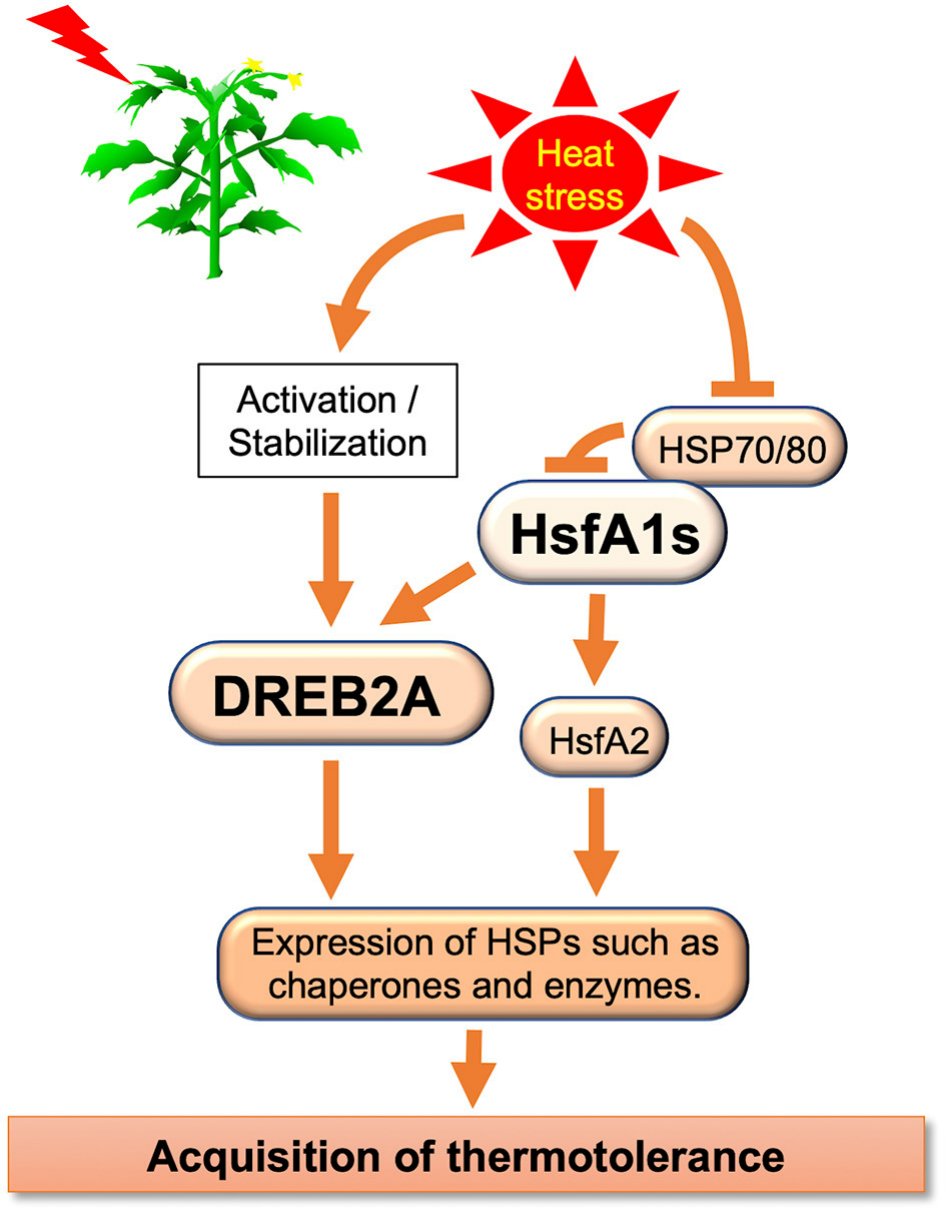
Schematic transcriptional regulatory network in plants examined in this review.

Plants respond to elevated temperatures and ensure survival through various mechanisms, such as transcription, translation, and regulation of calcium, phytohormone, sugar, and lipid signaling, and of primary and secondary metabolism (Bita and Gerats, 2013). Molecular pathway-related thermotolerance has been identified in *Arabidopsis*, tomato, and other species (Qu et al., 2013; Ohama et al., 2017) (Table 1). The complex transcriptional pathways were reviewed by Ohama et al. (2017). The HS factor (Hsf) is a transcription factor (TF) associated with HS (Figure 2). Many eukaryotes have one to three Hsfs, but plants have over 20, which are classified as A, B, and C. Class A Hsfs are transcriptional activators. Ikeda et al. (2011) found that class B Hsfs of *Arabidopsis*, HsfB1, and HsfB2b, are transcriptional repressors that negatively express heat-induced Hsfs (HsfA2, HsfA7a, HsfB1, and HsfB2b) and a few heat shock protein genes. Yoshida et al. (2011) analyzed the dehydration-responsive element-binding protein 2A (DREB2A) promoter and discovered a heat shock element that functions as a cis-acting element in the expression of HS responsiveness of DREB2A. They generated multiple mutants and found that HS-responsive expression of DREB2A was abolished in the *hsfa1a/b/d* triple and *hsfa1a/b/d/e* quadruple mutants. They further showed that HsfA1a, HsfA1b, and HsfA1d function as major positive regulators of HS-responsive gene expression and that four HsfA1-type proteins are important for gene expression during normal plant growth. Therefore, HsfA1 is the master regulator of the plant's HS response (HSR). Due to HS, HsfA1 causes a transcription cascade composed of many TFs. Higashi et al. (2013) reported HsfA1d, a protein identified through full-length cDNA Over-eXpressing gene (FOX) hunting, using *Thellungiella salsuginea*, a species closely related to *Arabidopsis*. cDNAs improve heat tolerance by regulating HS-responsive gene expression. Ohama et al. (2016) reported that the central region of HsfA1d, one of several *Arabidopsis* HsfA1, is an important regulatory domain that suppresses HsfA1d transactivation activity by interacting with heat shock protein70 (HSP70) and HSP90. They designated this region as the temperature-dependent repression (TDR) domain. Overexpression of constitutively active HsfA1d, which lacks the TDR domain, induced the expression of heat shock proteins in the absence of HS, thereby conferring strong thermal stability to the overexpressors. In this manner, HsfAs control many HS-related factors, including DREB2, and the understanding of their temperature-controlled mechanism is also progressing.

Four types of HsfA1 were isolated in *Arabidopsis* (HsfA1 a, b, c, and d) (Liu et al., 2011), while four different types were identified in tomatoes (HsfA1, a, b, c, e) (El-Shershaby et al., 2019). In HsfA1 families, HsfA1a seems to have a unique function as a master regulator for acquired thermotolerance, and it cannot be replaced by other genes (Mishra et al., 2002; Scharf et al., 2012). Other members of the HsfA1families are induced in specific tissues and stages of the HSR (El-Shershaby et al., 2019). SlHsfA1a function was confirmed by the heat tolerance levels at the incorporation of two HsfA1 transgene cassettes, resulting in a 10- to 15-fold increase in the overexpression line that contained a single HsfA1a transgene cassette and co-suppression line with two cassettes of transgene tandem inverted repeat inserted, respectively (Baniwal et al., 2004). Moreover, with a low abundance of mRNA, HsfA1a was constitutively expressed (Fragkostefanakis et al., 2016). HsfA1d increases heat tolerance in soybean (Ohama et al., 2017). HsfA1b is a member of the HsfA1 subfamily, which is induced under HS conditions (above 35°C). Under HS conditions, HsfA1a was stably expressed, whereas HsfA1b showed high variation in gene expression in mature green fruits and young leaves (El-Shershaby et al., 2019). In addition, the HsfA1b function is mainly controlled at the transcriptional level by HsfA1 members (Fragkostefanakis et al., 2016). HsfA1b was strongly expressed in fruits, and high fluctuation was observed among different tissues. The results suggested that HsfA1b is a later response gene under HS (El-Shershaby et al., 2019). Several tolerant genes, such as HsfA2, HsfA3, induced-heat shock protein HSPs, ET-responsive transcriptional coactivator multiprotein bridging factor ER24 (*LeMBF1*), cytosolic ascorbate peroxidase 3 *(SlAPX3)* (a ROS scavenger), and calcium-dependent protein kinase 2 (*CDPK2*), were isolated from the anthers (Frank et al., 2009; Zinn et al., 2010). Recently, Balyan et al. (2020) have reported that there is redundancy in cultivar-specific HS regulation compared to transcriptomes between resistant (CLN1621L) and susceptible (CA4) cultivars. Enzymes and proteins related to plant defense and abiotic stress are antagonistically expressed. This study suggested that three ET-responsive TFs (ERF.C1/F4/F5), as several novel HS-resistant genes, improved tomato HS resistance. Rao et al. (2021) reported HSFA7, HSFA6b, HSFA4c, HSFB1, and HSFB2b as new downstream targets of HSFA1a in tomatoes during **HS**.

Heat shock proteins are regulated by HSFs, which control protein quality (Scharf et al., 2012). HSPs are crucial chaperone proteins that are induced during HSR. The HSP family includes a number of small HSPs (sHSPs) and sub-family proteins HSP60, HSP70, HSP90, and HSP100 (Kotak et al., 2007; Qu et al., 2013). The *Hsp21* gene is related to chloroplasts and photosynthesis (Neta-Sharir et al., 2005; Zhong et al., 2013), whereas *HSP101* is among tolerant genes, such as stable Rubisco isoforms and other genes identified from anther profiling (Zinn et al., 2010). In HS, HSPs play important roles in the regulation of protein quality through protein denaturation. HSP21 is a small HSP in *Arabidopsis*, necessary for chloroplast development to protect photosynthesis (Zhong et al., 2013). HSP101 functions as a chaperone in protein degradation (Wang et al., 2004). Despite the important role it plays in sHSP thermotolerance, the underlying mechanisms are not known (Ohama et al., 2017).

Studying the expression levels of TFs and HSPs in tomatoes under HS will help understand the molecular mechanisms of mutant response to high temperatures.

## Influence of HS on the Reproductive Organs and Reproductive Phase in Tomato Plants

The reproductive stage of the plant and the reproductive organs are highly sensitive to HS, which is a major yield-reducing factor. Various reproductive phases, especially stages including meiosis in both male and female organs, pollen germination, pollen tube growth, pollen/pistil interactions, fertilization and post-fertilization processes, formation of the endosperm, and embryo development, are highly sensitive to HS (Warrag and Hall, 1984; Monterroso and Wien, 1990; Peet et al., 1998; Erickson and Markhart, 2002).

Tomato is an autogamous species with a flower structure that is compatible with self-pollination; the anther cones (stamens) cover the style (stigma or pistil). The position and maturity of the male (anther cone) and female (style) organs are markedly affected by various abiotic stresses, including HS during bud development, causing stigma (style) exertion in tomato flowers (Figure 1) (Saeed et al., 2007; Yan et al., 2009; Pan et al., 2019). The effects of elevated temperatures on tomato flower morphology have been previously explored. The tomato stigma under HS is exerted, preventing self-pollination, and causing fruit-setting failure (Sato et al., 2006; Giorno et al., 2013). The exertion of tomato stigmas induced by HS is associated with various factors and pathways, such as JA signaling (Pan et al., 2019). Pan et al. (2019) reported that stigma exertion induced by HS was a result of the higher susceptibility of the stamen to HS as compared to the pistil and the differences in cell morphology in both. The discrepant coregulation of pectin, sugar, expansion, and cyclin in stamens and pistils determined cell shape and number by regulating cell expansion and division under HS. Auxin is required to regulate high temperature-induced growth inhibition in both stamens and pistils. JA plays a crucial role in protecting pistils against HS, and the JA/JA receptor CORONATINE-INSENSITIVE 1 (COI1) signaling pathway is a key hub in stigma exertion. Müller et al. (2016) reported that concurrent reduction in pollen viability and pistil-like aberrant formation of anthers under HS is caused by altered localization of two pistil-specific gene products, *TRANSMITTING TISSUE SPECIFIC (TTS)* and *TOMATO AGAMOUS LIKE11 (TGL11)*. This is accompanied by reduced expression of B-class genes, such as *TOMATO APETALA3 (TAP3), TOMATO MADS BOX GENE6 (TM6)*, and *PISTILLATA* (*PI*) in the anthers (Kramer et al., 1998; Busi et al., 2003; de Martino et al., 2006). These reports showed that the downregulation of tomato B-class genes, induced by HS, contributes to anther deformation and reduced male fertility (Müller et al., 2016). Thus, flowers exposed to HS showed negative effects at various developmental stages, such as the inhibition of pollen release from anthers due to the failure of anther dehiscence, stigma exposure due to decreased stamen length, and pistil hyperplasia (Takeoka et al., 1991; Sato et al., 2002, 2006).

**Figure 3:**
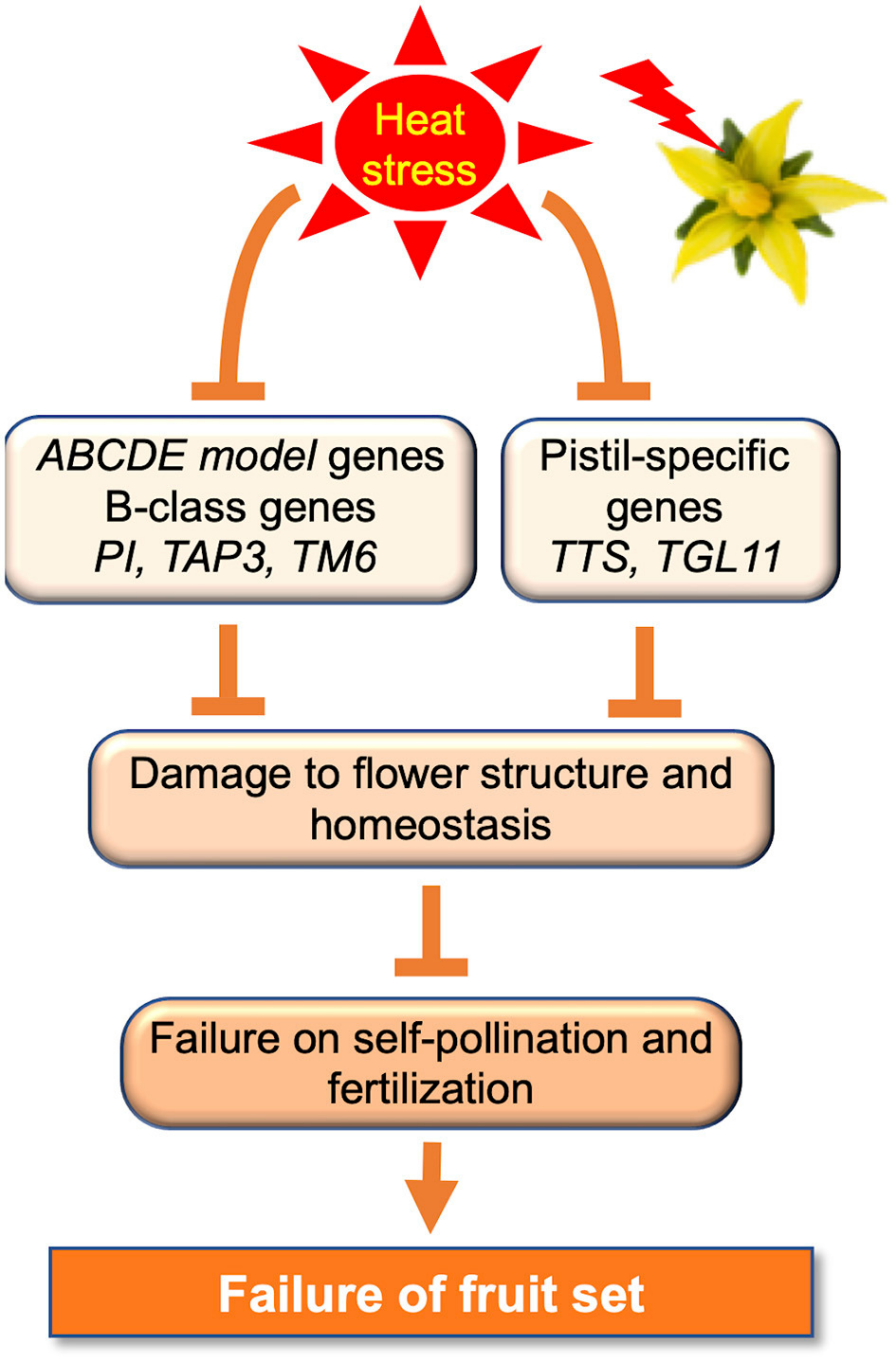
Gene regulation to avoid adverse effects of heat stress on the reproductive phase in tomatoes.

Maintaining floral morphological homeostasis is important because HS has adverse effects on flower morphology (Figure 3). The *ABCDE* model genes include five classes, A, B, C, D, and E of floral development, which is encoded using MAD-box TFs (Rijpkema et al., 2010; Smaczniak et al., 2012), with the exception of class A gene *APETALA2* (*AP2*) (Jofuku et al., 1994). In *Arabidopsis, AP1* belongs to class A, *AP3* and *PI* belong to class B, *AGAMOUS* (*AG*) belongs to class C, *SEEDSTICK* (*STK*), *SHATTERPROOF1* (*SHP1*), and *SHP2* belong to class D; and *SEPALLATA1* (*SEP1*), *SEP2, SEP3*, and *SEP4* belong to class E (Wellmer et al., 2014). In addition, the *AGAMOUS-LIKE 6* (*AGL6*)-clade genes *AGL6* and *AGL13* play crucial roles in floral organ development, especially in ovule formation (Murai, 2013). In tomatoes, under mild HS, expression of the B-class *PI, TAP3*, and *TM6* genes is reduced in the anthers (Müller et al., 2016). TM6 was partially silenced in response to temperature elevation, resulting in a reduced frequency of pistilloid anthers, pollen viability, and pollen quantity. Müller et al. (2016) suggested that downregulation of tomato B-class genes is related to anther deformations and reduces male fertility.

The *AGL6*-clade did not belong to the conventional *ABCDE* model genes that regulate floral structure in plants but is likely to play a role in the ovary formation (Schauer et al., 2009). In tomato plants, the *AGL6* mutant is related to fruit parthenocarpy (Klap et al., 2017). In addition, *AGL6* also acts as a key regulator of the transition between the state of “ovary arrest” imposed toward anthesis and the fertilization-triggered fruit set (Klap et al., 2017). Silencing *AGL6* results in green petals and fused sepals (Yu et al., 2017). The no apical meristem (*NAM*) protein is involved in the separation between sepal boundaries and flower whorls (Hendelman et al., 2013).

The reproductive phase of tomato plants starts from the first bud formation with the development of pollen that is more HS sensitive than female gametophytes and other vegetative organs (Bokszczanin et al., 2013). There are some reports on the pollen viability of tomato plants under HS, and flower buds at 7–15 days before anthesis were the most heat-sensitive of all developmental stages in tomato plants, as spindle formation in the meiosis phase is hypersensitive to HS (Sato et al., 2006). When pollen mother cells in the meiosis phase are damaged by HS, the quality and quantity of pollen grains are markedly reduced. As a matter of fact, tomatoes grown under 32/26°C day/night temperature could not release enough pollen, resulting in diminished fruit set (Sato et al., 2000, 2006). Additionally, pollen viability in tomato plants is controlled by a series of factors that are directly or indirectly involved in pollen thermotolerance. For example, secondary metabolites, such as flavonoids, accumulated in the mature pollen, might reduce the damage caused by ROS scavengers (Paupière et al., 2017). HS negatively affected both the early and late stages of pollen development. A complex network of metabolites and plant hormones is involved in the thermotolerance machinery of tomato pollen at different stages: (i) the early stage of pollen development involves the accumulation of unfolded protein response (UPR) in the endoplasmic reticulum (ER), cytoplasm, changes in histones, alternative splicing, ROS homeostasis, metabolic reprogramming, carbohydrates, plant hormones, and gibberellins (GAs); and (ii) the later stage of pollen development involves UPRs, ROS, amino acids (proline), carbohydrates, auxins, polyamines, flavonoids, and plant hormones (such as JAs, ETs, BRs, and ABA). Furthermore, compatible stigma and pollen also contributed to successful fruit and seed formation (Raja et al., 2019). Therefore, these factors should be considered when developing strategies to improve tomato fruit production under high-temperature conditions.

## Negative Effects of HS on Fruit Development in Tomato Plants

Heat stress suppressed tomato fruit development, resulting in abnormal fruit shapes and negative changes in color processing. HS not only decreases fruit setting but also influences fruit dehiscence and fruit morphology, resulting in dehydration, with wrinkled skin and dry, locular fruit cavities (Lin et al., 2011). Fruit quality is controlled by the cell number and cell size, sugar accumulation, traits related to fruit shape, colorimetry, total solids, texture, and flavor (Cheniclet et al., 2005; Chusreeaeom et al., 2014; Quinet et al., 2019). Fruit size is determined by the coordinated control of cell division and cell expansion. Fruit size is regulated by several molecular mechanisms, including hormonal regulation, the CLAVATA-WUSCHEL (CLV-WUS) signaling pathway, the MADS-box family, the ubiquitin-proteasome pathway, quantitative trait loci (QTLs), microRNA, and endoreduplication (Yuste-Lisbona et al., 2020; Zhao et al., 2021). The CLV-WUS signaling pathway regulates the maintenance of stem cells in shoot and floral meristem, contributing to several agronomic traits, such as flower and fruit numbers (Fletcher, 2018; Quinet et al., 2019). The CLV-WUS feedback loop regulates meristem activity and floral meristem size during the initial phase of tomato fruit development, and it determines carpel number in flowers and, thus, seed locules in fruit during the later phases (Rodríguez-Leal et al., 2017). The signaling peptide CLV3 interacts directly with CLV1 or CLV2, which are leucine-rich repeat receptor kinases, and activates a signaling cascade that negatively regulates the activation of the stem cell-promoting TF WUS (Somssich et al., 2016). A loss-of-function mutation in any *CLV* genes, such as natural mutations in *fasciated (fas)* and *locule number* (*lc*), results in increased proliferation of stem cells and consequently the development of extra floral organs and larger fruits (Barrero et al., 2006; Xu et al., 2015; Fletcher, 2018). Jones et al. (2021) reported that high temperatures bypass CLV signaling and upregulate auxin through transcriptional repressor EARLY FLOWERING 3 (ELF3) in *Arabidopsis*; therefore, high temperatures and ELF3 regulate auxin-dependent primordia production. However, it is unclear how CLV2/CRN is involved in auxin-dependent flower initiation in *Arabidopsis*. Additionally, auxin and GA signaling pathways stimulate and directly activate tomato fruit sets (de Jong et al., 2009). *TOMATO AGAMOUS1 (TAG1)* and *ARLEQUIN/TOMATO AGAMOUS LIKE1 (TAGL1)* genes, which are members of the tomato MADS-box gene family, influence fruit size in tomatoes (Gimenez et al., 2016). Tomatoes that overexpress *ZjDA3*, an ortholog of *Arabidopsis* ubiquitin-specific protease (DA3/UBP14) in Chinese jujube (*Ziziphus jujuba* Mill.), have reduced fruit size and weight (Guo et al., 2021). The level of endoreduplication in tomatoes was correlated with cell size in fruit pericarp (Cheniclet et al., 2005). The expression of *CCS52A* (*Cell cycle switch protein*) or *WEE1* (cell cycle-associated protein kinase) genes involving endoreduplication in tomatoes affects cell size and fruit size (Gonzalez et al., 2007; Mathieu-Rivet et al., 2010). Although it is known that miRNA172 influences fruit size regulation in horticultural plants, such as tomato and apple (José Ripoll et al., 2015; Yao et al., 2016), their relationship in tomatoes under **HS** is unclear. Four genes are related to fruit shape in tomatoes; *FASCIATED (FAS)* and *LOCULE NUMBER (LC)* control flattening and fruit locule number and *SUN* and *OVATE* contribute to fruit elongation (Rodríguez et al., 2011). HS increases parthenocarpic fruit production (Pan et al., 2017; Xu et al., 2017; Shinozaki et al., 2018; Pham et al., 2020). Parthenocarpic fruits are induced by several plant hormones, such as GAs and auxin (Ariizumi et al., 2013; Bita and Gerats, 2013; Shinozaki et al., 2020). Additionally, ABA plays a critical role in regulating transcript expression to induce plant defense responses under HS (Scharf et al., 2012; Paupière et al., 2017; Rieu et al., 2017).

In tomatoes, sugar content is closely linked to fruit development (Kanayama, 2017) and is controlled by the *phosphoenolpyruvate carboxykinase* gene (*PEPCK*) (Huang et al., 2015), biochemical factors (Beckles et al., 2012), vacuolar processing enzymes (Ariizumi et al., 2011), and putative sucrose sensors (Barker et al., 2000). Total soluble solid (TSS or Brix°) represents the fruit sugar content. TSS content is highly influenced by various biotic and abiotic stresses, including HS, which also damages fruit morphology and quality. Tomatoes have three different developmental stages (Ho, 1996): (i) cell division to increase cell number that contributes to mature fruit size, (ii) rapid cell expansion, and (iii) fruit ripening (Ezura, 2016). Sugar accumulation also generally consists of three steps: first, the vascular system imports the sucrose and water influx; second, starch biosynthesis and sugar metabolism; and third, the breakdown of starch into glucose while fruits soften rapidly (Carrari et al., 2006). In the TSS of tomato fruits, sugars (glucose, galactose, and fructose) contributed the largest portion (Selahle et al., 2014), and TSS commonly ranges from 4 to 6 °Brix among different genotypes.

In tomatoes, the functional amino acid polymorphism of cell wall invertase (*LIN5*) was encoded by *Brix9-2-5*, which regulates fruit sugar content (Fridman et al., 2000, 2004). Fruit sugar content and seed development are affected by the inhibition of sucrose transporters. Several sucrose transporters (*SUT* or *SUC*) that are essential membrane proteins localized in the phloem sieve element, including *LeSUT1*, are expressed in leaves; *LeSUT2* is expressed in stems, fruits, and anthers, and *LeSUT3* is expressed in ovaries and immature fruits (Barker et al., 2000; Weise et al., 2000; Hackel et al., 2006). In addition, five genes encode vascular processing enzymes *(SlVPEs)*: two seed coating type genes, *SlVPE1* and *SlVPE2*, one seed type gene *SlVPE4*, and two vegetative genes, *SlVPE3* and *SlVPE5* were reported (Ariizumi et al., 2011). *SlVPEs* are negative regulators of sugar content in tomato plants. Therefore, using transgenic RNAi lines for single or multiple gene expression could be an approach to increase sugar accumulation in tomatoes.

## Breeding Materials and Technology to Mitigate HS Influence

The morphological and physiological traits in the vegetative and reproductive phases, that are useful for identifying heat-tolerant tomatoes, are summarized in Figure 1. Several breeding lines have been recently identified and evaluated by focusing on phenotypes and indicators to generate a heat-tolerant tomato (Table 2). Zhou et al. (2015) described the differences in the quantum efficiency of photosystem II (F*v*/F*m*) between the heat-tolerant and heat-sensitive groups of tomato entries, and they reported that F*v*/F*m* was useful as an early indicator of HS tolerance. Subsequently, Poudyal et al. (2018) evaluated some genotypes using F*v*/F*m* and identified some novel heat-tolerant entries. Paupière et al. (2017) evaluated the accessions of 17 different cultivated and wild tomato phenotypes to high temperatures, focusing on a pollen viability screening approach, and identified thermotolerant and thermosensitive entries. Some heat-tolerant tomato mutants were identified from over 4,000 lines of Micro-Tom tomato mutant collections by evaluating pollen viability, fruit yield, and fruit setting under long-term HS (Pham et al., 2020). Kugblenu et al. (2013) evaluated heat adaptation traits, such as flower drop and fruit number using commercial varieties, that are widely available to farmers in West Africa. Under HS, tomato plants carry the *procera (pro)* and *procera-2 (pro-2)* mutants, which are loss-of-function mutants of tomato *DELLA (SlDELLA)*; the hypomorphic allele showed higher fruit set efficiency, and their fruits were parthenocarpic (Shinozaki et al., 2018). In general, there are two main approaches to studying thermotolerance in tomato plants: screening the germplasm (for long-term mild heat treatment) and physiological responses (for short-term heat shock, up to 45°C). Therefore, breeding and genetic engineering strategies can be individually applied or suitably integrated to develop HS tolerant lines or mitigate the effects of HS on tomatoes. Meta-quality trait loci (meta-QTL analysis) and multi-parent advanced generation intercross (MAGIC) have been used to provide a higher mapping resolution in heat-tolerant tomato breeding programs. In addition, speed breeding and genomic selection (GS) significantly contribute to thermotolerance in tomatoes (Ayenan et al., 2019; Aleem et al., 2020; Bineau et al., 2021). There are several ways of mitigating the effects of **HS** on tomatoes, for example, applying plant growth-promoting rhizobacteria (PGPR) (Mukhtar et al., 2020), or using 6 ppm sulfur (Ali et al., 2021), or nitrate seed priming (Kumar V. et al., 2021). These breeding materials can be used to elucidate the physiological responses conferring adaptation to HS and provide a basis for further studies on the identification of heat-tolerant lines and phenotyping segregating populations.

**Table 2:** Heat-tolerant tomato germplasms and screening conditions.

**Identified tolerant accessions or genotype**	**Screening environment**	**Screening conditions**	**Traits and phenotypes**	**References**
LA1500, LA1563, LA1994, LA2093 (*S. pimpinellifolium*), LA3120, LA3183, Bush Italian Roma, Super Sweet	Controlled environment Open field (for validation)	67 genotypes, 2 heat tolerant and 2 heat sensitive for validation HS: 36/28°C for 4 d 67 genotypes HS: 40°C for 7 h	Pollen germination rate Pollen tube length Fruit set	Zhou et al., 2015
Doti Local 1, HRD 1, HRD 17, ST 10, ST 52	Controlled environment Open field	HS: 4 d at 38/28°C, 5 d for recovering 38/26°C	Photosynthesis Stomatal conductance Plant size Pollen viability Fruit yield	Poudyal et al., 2018
LA2854, LA1478, Nagcarlang, CL5915-153D4- 3-3-0, CL1131-0-0-13-0-6, CLN1621F and CL5915-93D4-1-0-3 (highest pollen viability) LA1580, LA2854 (*S. pimpinellifolium*), CLN1621F, CL5915-206D4-2-2-0-4, CLN65-349D5-2-0, M-82, CL5915-93D4-1-0-3 (highest number of pollen per flower)	Controlled environment	HS: 32/26°C (day/night) Control: 25/19°C (day/night under 12-18 h of natural day light for 1 month)	Number of pollen per flower Pollen viability	Paupière et al., 2017
15 heat-tolerant tomato mutants	Controlled environment Greenhouse	HS: 35/25°C, 16 h/8 h light/dark, 60.0 μmol m^−2^ s^−1^ Control: 25°C, 16 h/8 h light/dark, 60.0 μmol m^−2^ s^−1^ Greenhouse: Over 35°C (daily maximum temperature)	Flower number Fruit number Fruit set Fruit yield Average fruit weight Seed number SPAD score Pollen viability Pollen germination	Pham et al., 2020
Nkansah (CLN2001A) (high fruit set)	Controlled environment	HS: 33.8/25.9°C (day/night)	Percentage of flower drop Number of fruits Days to flowering Fruit yield per plant Fruit weight Number of truss Number of flowers per truss Number of fruits per plant	Kugblenu et al., 2013
*procera (pro), procera-2 (pro-2)*	Greenhouse	Summer conditions (June- September 2014)	Fruit number Fruit set Fruit yield Average fruit weight Stem elongation Brix value	Shinozaki et al., 2018
69 genotypes (13 and 19% of the core collection and MAGIC populations, respectively)	Greenhouse	MAGIC HS: 26.9/34.4°C Control: 21.2/28.8°C Core collection HS: 27.5/35.5°C Control: 22.7/31°C Daily mean/maximal temperatures	Stem diameter Leaf length Plant height Flowering time Flower number Fruit number Fruit set Average fruit weight Fruit color Fruit pH	Bineau et al., 2021

On the other hand, genetic modification (GM) technology provides rapid and effective cultivars exhibiting tolerance to diverse abiotic stresses, including HS, compared with traditional breeding. There has been some research on the supply of heat tolerance using various genes involved in the regulatory and signaling pathways (Gerszberg et al., 2015) (Table 3). To date, the provision of tolerance to HS was performed using transgenic plants overexpressing TF or HSP involved in transcription regulatory networks, such as *HsfA1* (Mishra et al., 2002), *hsp21* (Neta-Sharir et al., 2005), *MasHSP24.4* (Mahesh et al., 2013), and *MT-sHSP* (Nautiyal et al., 2005). Transgenic plants expressing yeast S-adenosyl-l-methionine decarboxylase (*SAMDC*), which is a key regulatory enzyme in polyamine biosynthesis, increased the accumulation of spermidine and spermine and enhanced antioxidant enzyme activity, thereby protecting membrane lipid peroxidation. Subsequently, the plant was protected from HS by improving the efficiency of CO_2_ assimilation through its enhanced activity and protection (Cheng et al., 2009). Transgenic tomato with an increased anthocyanin-associated R2R3-MYB TF, *Lycopersicon esculentum Anthocyanin 2* (*LeAN2*) overexpression, is highly tolerant to HS (Meng et al., 2015). LeCDJ1 *(Lycopersicon esculentum* chloroplast-targeted DnaJ protein) is involved in the plant response to ABA. *LeCDJ1* overexpressed plant improved growth, chlorophyll content, lower malondialdehyde accumulation, relative electrical conductivity, and less PSII photoinhibition under HS (Kong et al., 2014). Transgenic tomato plants overexpressing choline oxidase (COD), which is involved in glycine betaine (GB) synthesis, showed a high accumulation of GB. The *codA*-transgenic plants showed increased CO_2_ assimilation and photosystem II photochemical activity and mitigated the accumulation of H_2_O_2_, superoxide anion radicals, and malondialdehyde. Zhang et al. (2020) suggested the major role of GB in HS tolerance and the importance of H_2_O_2_ as a signaling molecule in heat resistance.

**Table 3:** Representative genes available for improving heat tolerance in tomato plants.

**Gene/locus symbol**	**Source**	**Expression**	**Defined function**	**References**
*HsfA1*	Tomato	Overexpression	Transcription regulatory network	Mishra et al., 2002
*hsp21*	Tomato	Overexpression	Accumulation of heat shock proteins Transcription regulatory network	Neta-Sharir et al., 2005
*MasHSP24.4*	*Musa acuminata*	Expression	Accumulation of heat shock proteins Transcription regulatory network	Mahesh et al., 2013
*MT-sHSP*	Tomato	Expression	Accumulation of heat shock proteins Transcription regulatory network	Nautiyal et al., 2005
*SAMDC*	Yeast	Overexpression	Polyamine biosynthesis	Cheng et al., 2009
*LeAN2*	Tomato	Overexpression	Anthocyanin-associated R2R3-MYB transcription factor	Meng et al., 2015
*LeCDJ1*	Tomato	Overexpression	Chloroplast-targeted DnaJ protein	Kong et al., 2014
*codA*	Tomato	Overexpression	Glycine betaine (GB) synthesis	Zhang et al., 2020
*slmapk3*	Tomato	CRISPR/Cas9	Mitogen-activated protein kinases (MAPKs) family	Yu et al., 2019

## Developing Heat-Tolerant Tomatoes for Breeding

Genome analysis has progressed significantly and allows us to breed genomes not only in major crops, such as rice but also in vegetables, such as tomatoes. Large-scale phenotypic analysis has also seen significant development. To make the best use of these technologies, it is important to choose traits carefully and conduct the evaluation that suits the objectives, in an appropriate environment. In tropical regions, such as sub-Saharan Africa (SSA) and Southeast Asia, where rapid population growth is predicted in the future, sustainable production and supply of vegetables will contribute to food security, household improvement of farmers, nutrition improvement of residents, and health promotion. However, currently, most of the vegetables in the tropics are produced and consumed as it is. The primary varieties cultivated are developed by foreign seed companies in developed countries, and these varieties are not very resistant to high temperatures and humidity. Insufficient resistance to diseases results in instability in the yield and quality. Our research team is promoting genome breeding research to develop vegetables, such as tomatoes that are resistant to high-temperature stress, by utilizing unused genetic resources with excellent traits and making full use of marker selection based on genomic information, especially in SSA and Southeast Asia.

In vegetable breeding, in addition to genome breeding that makes full use of genome information and phenotyping technology, there is great potential for genome editing that has been developed in recent years. In countries with a product-based mindset, unlike traditional GM crops, GM technology is used to generate genome-edited crops, but null segregants do not contain transgenes, which can be suggested using Southern hybridization and PCR (El-Mounadi et al., 2020; Kumar S. et al., 2021). In this case, the deregulation process required for GM crops becomes unnecessary; thus, commercialization is relatively easy, and consumers' resistance to GM can be excepted. The University of Tsukuba leads the development and sale of the tomato variety Sicilian Rouge High GABA (Sanatechssed; https://sanatech-seed.com/en/), which has improved GABA contents (a component that has the effect of suppressing blood pressure rise) through genome editing (Nonaka et al., 2017; Lee et al., 2018; Yamamoto et al., 2018; Gramazio et al., 2020). To date, many genes involved in HS resistance have been identified. It is expected that the improved genome editing technology will be used to improve HS resistance by inducing mutations in the negative regulatory genes, which are the key to HS resistance in vegetables, such as tomatoes. Genome editing has already been used to improve stress resistance in tomatoes. For example, the *mitogen-activated protein kinase* (*mapK*) *3* gene, *slmapK3* gene, branched-amino acid (ALS1), cytidine base editor (CBE*)* genes, and LATERAL ORGAN BOUNDARIES DOMAIN—LBD TF gene—*SlLBD40* increased resistance to HS, sulfonylurea herbicide chlorsulfuron, and drought, respectively (Ayenan et al., 2019; Yu et al., 2019; Salava et al., 2021; Xia et al., 2021). Both the achievements of genome editing technology with regard to tomatoes and the identification of key genes, such as *HsfA2, HsfB1, JA/COI1, SlAGL6*, and *SlIAA9*, are related to the thermotolerant acquires mechanism in tomatoes, positively contribute to the breeding of heat-tolerant tomato. On the other hand, considering the future movement of products across countries, harmonization between countries that handle genome editing on a product basis and countries that handle genome editing on a process basis is a future concern. Genome editing is an epoch-making technique that can easily cause mutations that occur in nature for the chosen study species. However, there is a concern that consumers will reject it simply because it is a new technology that manipulates the genome. Scientists need to obtain scientific evidence and communicate it to society by communicating closely with governments, producers, consumers, the media, and other stakeholders.

It is necessary to elucidate the physiological mechanisms underlying heat tolerance and facilitate breeding research to improve both tolerance and recovery ability with respect to resilience. Interdisciplinary approaches that go beyond genetic breeding, such as improving HS resistance by utilizing plant-microbial interactions by elucidating the relationship between the microbiome and HS, may also be effective. By utilizing Digital Transformation (DX), which has developed remarkably in recent years, from the viewpoint of Genotype x Environment x Management (G x E x M), to elucidate the appropriate combination of excellent varieties (genotype), cultivation environment and method (management), model and recommend it to agricultural sites. Utilizing these various innovations may improve climate change adaptation in vegetables, such as tomatoes, by improving HS tolerance in a broad sense.

## Conclusion

The latest IPCC report clearly indicates that climate change is currently occurring and will threaten food security in the future. The increasing vulnerability of future food systems is a point of concern. **HS**, resulting from climate change-induced temperature increase, has a negative impact on all stages of crop growth. For fruiting vegetables, such as tomatoes, even moderate HS reduces fruit set and quality; therefore, enhancing crop HS tolerance is among the best ways to adapt to climate change. In this review, we discuss the important processes that affect the growth and yield of tomatoes, especially HS. This review examines the molecular, morphological, and physiological mechanisms that contribute to HS tolerance and the challenges of developing thermostable vegetable varieties. HS has several complex adverse effects on a wide range of plant growth stages in tomatoes. To understand plant tolerance mechanisms against HS, it is necessary to investigate molecular tolerance mechanisms at each growth stage and type of HS (short or long term). There are several reports on gene regulation networks with respect to short-term HS, but there are few regarding long-term HS. Considering the need to produce heat-tolerant tomato plants, it is crucial to determine how HS occurs in each target area, select germplasm for screening heat tolerance materials, and design molecular pathways to adjust to the target. The nutritional and functional properties of vegetables, including tomatoes, are valuable in terms of global food and nutritional safety assurance. Studies investigating the rapidly increasing HS tolerance and the development of heat-resistant vegetable varieties will contribute toward climate change adaptation and the construction of sustainable and resilient food systems to achieve sustainable development goals.

## Author Contributions

KH and KN wrote the draft of the manuscript. All authors contributed to manuscript revision, read, and approved the submitted version.

## Conflict of Interest

The authors declare that the research was conducted in the absence of any commercial or financial relationships that could be construed as a potential conflict of interest.

## Publisher's Note

All claims expressed in this article are solely those of the authors and do not necessarily represent those of their affiliated organizations, or those of the publisher, the editors and the reviewers. Any product that may be evaluated in this article, or claim that may be made by its manufacturer, is not guaranteed or endorsed by the publisher.

## Abbreviations

ABA: abscisic acid
*AGL6*: AGAMOUS-LIKE 6
*AP3*: APETALA3
BRs: Brassinosteroids
DREBs: dehydration-responsive element-binding
ER: endoplasmic reticulum
ET: ethylene
GAs: gibberellins
GHG: greenhouse gas
HS: heat stress
HsfA1: heat stress transcription factor A1
HSFs: heat stress transcription factors
HSPs: heat shock proteins
JA: jasmonate
PCD: programmed cell death
*PI*: PISTILLATA
ROS: reactive oxygen species
*sHSPs*: small heat shock proteins
SA: salicylic acid
*TM6*: TOMATO MADS BOX GENE6
TSS: total soluble solids content
UPR: unfolded protein response.
